# The Need for an Alternative Health Claim Process for Foods Based on Both Nutrient and Contaminant Profiles

**DOI:** 10.1016/j.cdnut.2024.103764

**Published:** 2024-04-25

**Authors:** Konstantinos Christos Makris, Michael Chourdakis

**Affiliations:** 1 Cyprus International Institute for Environmental and Public Health, School of Health Sciences, Cyprus University of Technology, Limassol, Cyprus; 2 Laboratory of Hygiene, Social & Preventive Medicine and Medical Statistics, Faculty of Health Sciences, Aristotle University of Thessaloniki, Thessaloniki, Greece

**Keywords:** health claim, food, risk assessment, food contaminants, pesticides, exposome, climate change

## Abstract

Most authorized health claims on foods have been established on the basis of single dietary components, mainly micronutrients, such as vitamins, minerals, and possibly bioactives. Failure to sufficiently define and characterize the nutritional profile of a food product is one of the main reasons for rejection or incomplete status for thousands of health claim applications, whereas the food’s contaminant profile is simply not accounted for. The objective of this work was to highlight the accumulating scientific evidence supporting a reform of the health claim evaluation process for foods toward more holistic approaches. This would entail the characterization of multiple nutrient-contaminant pairs and contaminant mixture profiles at contaminant levels currently considered “safe,” including their interactions that would impact human health outcome(s) in a net positive or negative direction. The notion of a stable nutritional profile in food commodities has been challenged by studies reporting a variable food contaminant content and a declining content of proteins/micronutrients in crops due to anthropogenic greenhouse gas emissions. A holistic approach in the health claim process for foods would entail the incorporation of cumulative risk assessment and/or risk-benefit protocols that effectively combine health risks and benefits associated with multiple nutritional and contaminant attributes of the food/diet under evaluation.

## Setting the Scene on Food Contaminants, Nutrients, and Health Claims

Τhe global goal to minimize the adverse impacts of synthetic chemicals by 2020 was not achieved [1,2]. The sound management of chemicals covering all stages of the life cycle is considered an essential component of the United Nations 2030 Agenda toward meeting the sustainable development goals (SDGs). Notwithstanding, synthetic chemical production is expected to double globally by 2030, whereas harm to people and the planet will likely exacerbate unless appropriate policy measures are set in place [1,2]. This is highly relevant for the agriculture and food sector in relation to the SDG target 2.4 of sustainable food production, the opportunity to scale up integrated pest management and agroecologic approaches, including the use of nonchemical alternatives as highlighted in the SDG 2030 Agenda [2]. Further, the European (EU) *Farm-to-Fork* strategy, the *Zero Pollution Action Plan*, the *Green Deal,* and the EU Chemicals Strategy for Sustainability set the regulatory framework for promoting sustainable and healthy food production, including the impact assessment of synthetic chemical mixtures on human health and the environment [3].

Regulatory action centered on evidence-based approaches to address consumer health concerns over the myriad of manmade chemicals that people are regularly exposed to via food may be urgently needed [4]. The Lancet Commission on Pollution and Health recognized chemical pollution as a great and growing global problem [4]. The EU Zero Pollution Action Plan set a target of a 50% reduction in the overall use of chemical pesticides and a 50% reduction in the use of more hazardous pesticides by 2030. Since the 1950s, the global market of >140,000 chemicals and pesticides, such as lead, polychlorinated biphenyls (PCBs), dichlorodiphenyltrichloroethane, “forever” chemicals (per- and polyfluorinated substances, PFAS), or emerging ones (glyphosate and endocrine disruptors) have been commonly detected in various parts of the food chain. A rigorous premarket toxicity/safety evaluation of new chemicals and herbicides/insecticides is not usually the norm globally [4]. Such policy gaps call for systemic interventions all the way from farm-to-fork if we were to achieve sustainable and healthy food ecosystems. The situation is perplexing if one considers the challenges of global pollution together with other key planetary health drivers, such as the climate crisis, land use change, or biodiversity loss that all impact key proximate environmental causes of disease (e.g., air quality, food quality, water quality/quantity, etc.) [5]. The planetary health framework integrates the interplay of sustainable food production systems together with global pollution challenges and global environmental change that may hold important ramifications for human health, depending on the influence of modifiers, such as education, culture, wealth, or food, environment, and health policies [[6], [7], [5]].

A sustainable food system is a food system that delivers food security, safety, and nutrition for all in such a way that the economic, social, and environmental bases to generate food security and nutrition for future generations are not compromised [8]. Food safety aspects are regulated by the General Food Law Regulation 178/2002 in Europe, promulgating that a food shall not be placed on the market if it is unsafe. Article 14 of the EU Food Law 178/2002 states that food shall be deemed to be unsafe if it is considered *1*) injurious to health and *2*) unfit for human consumption. It is the responsibility of the food industry/business to ensure the safety of produced foods, including legislative requirements for residues of plant protection products, or to bring forward an authorization request when a novel food is produced.

The proper evaluation and informative labeling of a food product with evidence-based health attributes represent an important component of a healthy and sustainable food system. The processes and criteria of labeling a food as “healthy” are governed by the instrumental EU Regulation 1924/2006, whereas the United States Food and Drug Administration has recently proposed the update of the definition for the implied nutrient content claim “healthy,” to be consistent with current United States nutrition science and dietary guidance [9]. According to the EU Regulation 1924/2006, a nutrition claim states, suggests, or implies that a food has beneficial nutritional properties, such as “low fat” or “high in fiber,” whereas a health claim states, suggests, or implies that health benefits can result from consuming a given food, such as “..helps maintain normal blood cholesterol levels.”

The EU Food Law allows for the classification of foods into safe and unsafe categories. However, gaps or differences in food safety assessment approaches may, at times, be associated with adverse health effects on consumers, calling for stronger regulatory harmonization at a global scale. At the population level, unhealthy dietary habits, such as the consumption of ultra-processed foods [10] and diets low in fruits and vegetables or fibers, have been associated with a higher burden of noncommunicable diseases (NCDs) [11,12]. Per the SDG target 3.4 to reduce premature mortality from NCDs by a third by 2030 relative to 2015 levels, dietary interventions (e.g., diets higher in fruits and vegetables, among others) are warranted to potentially prevent 1 in every 5 deaths globally [11].

Despite the diet-centered nature of population health risk factors in analyses for the global burden of disease, such as a diet low in vegetables/fruits [11,12], a nutricentric emphasis is typically put on health claims as described in the EU Regulation 1924/2006 (the nutrition and health claim regulation). This EU Regulation provides the criteria to label a nutrient/food product with established health benefits to human health. The European Food Safety Authority (EFSA) defines a health claim as “any statement on labels, advertising, or other marketing products that health benefits can result from consuming a given food (EU 1924/2006). For example, an authorized health claim refers to the health benefits of docosahexaenoic acid (DHA) maternal intake and its contribution to the normal development of the eye of the fetus and breastfed infants (EFSA, Q-2008-773). The need to move away from this nutricentric approach toward a holistic one was first brought forward by Scrinis [13] and later by others [[14], [15], [16]]. Scrinis [13] was perhaps the first to describe the nutritionism paradigm that was centered around a reductionist focus on single nutrients. A holistic approach would entail the consideration of foods rather than nutrients and dietary patterns rather than isolated food compounds [15]. The degree of food processing, which is evaluated using both metrics of food matrix and composition, is of importance in a holistic approach for evaluating nutritional benefits or risks in human health [14,16].

It appears that there is room for improvement in the current EU policy framework on health claims by considering multiple nutritional and contaminant attributes in foods instead of the current reductionist focus on the nutrient contents of the food under evaluation. This argument could further extend into the general food law system, as well, because it is well known that the current food law does not deal with the so-called “grey area foods” [17]. These gray area foods are defined as foods that do not pose a food safety risk in a legal sense, but they could pose a threat to human health because of other factors, such as their nutritional and/or contaminant composition [17].

Thus, the objective of this work was to highlight the regulatory and scientific evidence supporting a reform of the health claim evaluation process based on the integrated characterization of multiple nutritional and contaminant attributes of foods, moving away from the nutricentric dogma. Nevertheless, there are numerous ways to improve health claims toward a holistic methodological framework and novel assessment tools. The example of food contaminants mentioned in this work can be expanded to cover other chemicals, food additives and other food contaminants. The supporting scientific evidence and the means to operationalize an alternative health claim evaluation framework are presented below. The regulatory context of this work used the EU framework as an example, but the proposed reform in the health claim process relies on scientific evidence of global applicability.

## The Debate of Health Claims and Their Current Nutricentric Focus: The EU Case

In the EU Food Law, food safety issues are defined in terms of risk and met with stringent regulation, whereas nonsafety issues are primarily dealt with by providing food information to consumers to choose foods freely, yet responsibly. According to Articles 3(9) and (14) of Regulation (EC)178/2002, food safety assesses risk to biological, chemical, or physical hazards only, but without dealing with human health risk considerations. Human health aspects that relate to food consumption are absent from the current EU Food Law.

As such, the EFSA is not mandated to review safety considerations when it comes to scientifically assessing health claim dossiers, part of the EU Regulation 1924/2006. The EU Regulation 1924/2006 deals only with products that are either on the market already or have been authorized to be sold in the EU market. Health claims made on foods ensure that any health claim made on a food label in the EU is clear and substantiated by scientific evidence. For instance, the authorized health claim on linoleic acid clearly describes its contribution to the maintenance of normal blood cholesterol concentrations. The EU Regulation 1924/2006 binds the EFSA with specific rules and criteria to evaluate incoming health claim dossiers. These EFSA rules and criteria are largely based on the following set of key questions: *1*) Is the food product sufficiently defined and characterized? *2*) Is the claimed effect sufficiently defined, and is it a beneficial/physiological effect? *3*) Have pertinent human studies been presented to substantiate the claim? Following satisfactory responses, a comprehensive appraisal and integration of scientific evidence of all relevant studies with emphasis on randomized controlled trials, if any, is undertaken. The amount of the nutrient or food component in question, together with the composition of a particular food or food category, must be sufficiently defined in making accurate claims on food [18].

The value of health claims toward helping consumers make healthier food choices has been debated [19]. This has been largely attributed to the cognitive bias of a health halo effect where a health claim on a specific nutrient and its health attributes spill over to other nonclaimed qualities of the food under consideration [20]. For example, fast food restaurants leveraged healthy foods such as apples and milk to promote fast food via advertisements targeted at children [21]. A health halo effect would discourage consumers from seeking more information about the full nutritional value of a food that displays a health claim [19]. In countries with already high protein intake, the insistence on protein intake nutrition messages from protein sources linked with chronic diseases would have adverse public health consequences unless the food’s amino acid profile quality and its impact on human health and the environment are both highlighted [22].

Up to date, EFSA has evaluated thousands of health claim applications, and among them, >2000 applications have been considered either as complete but not authorized because of not satisfying the stringent evaluation criteria for health claims, or many of them are treated as incomplete, asking for more data before start-the-clock evaluation process is re-launched [23]. It appears that even if a cause-effect relationship has been preliminarily established, failure to sufficiently define and characterize the food product is one of the most frequent reasons for rejection/incomplete status for thousands of health claim applications [23]. As an example, insufficient food characterization was the main reason for the non-authorization of a proposed health claim on organic foods [24]. This nutricentric-based criterion does not allow EFSA to fully appraise the health evidence of the food/constituent under evaluation for such claim dossiers [23]. Some notable failing examples in the health claim evaluation process are, but are not limited to, the following: *1*) lack of nutritional information on key food components or categories of food under evaluation, *2*) focus on food chemical contaminants and pesticide residues or additives, and *3*) focus on foods with wide variability in specific nutrients and/or chemical contaminants or pesticide residues across food types. A major limitation of food characterization in the current health claim evaluation process is the fact that the absence or reduction of food contaminants in certain foods is not currently included in EFSA’s jurisdiction to characterize foods; this has been explained by the notion that a large variance in chemical contaminants and pesticides between-, and within-foods is anticipated [23,25].

Therefore, it follows that current health claim assessments on foods are conducted on the basis of the following: *1*) single nutrients, mainly micronutrients, such as vitamins, minerals, and possibly bioactives, and *2*) they do not consider the concomitant presence of food contaminants (e.g., chemicals and pesticide residues). The safety of chemical contaminants and pesticide residues in foods sold in Europe and in the United States is regulated via specific cutoffs of maximum contaminant/residue levels established by EFSA (EU 315/93, EU 2023/915) and the United States Environmental Protection Agency (with the aid of the United States Food and Drug Administration), respectively. The derivation of safe levels for food contaminants is based on the risk assessment for single chemicals, using the tolerable daily intake or the acceptable daily intake cutoffs. The current food safety assessment by the EFSA considers food contaminants (chemicals and pesticides), but it often relies upon the one-substance-at-a-time approach, evaluating the single contaminant hazards and the estimated risk based on exposures for different age groups. Under a strict food safety evaluation regime, the characterization and management of public health risks associated with chronic exposures to food contaminants, such as manmade chemicals (e.g., metals, dioxins, and mycotoxins) or pesticides are often disregarded as they don’t fit within the relatively narrow scope of the Food Safety Law system [17,26,27]. Nevertheless, strong or suspected links have been already documented for pesticides in foods and a suite of chronic diseases, such as non-Hodgkin lymphoma, multiple myeloma, ovarian, breast, brain, and prostate cancers, or neurologic disorders, such as Parkinson’s and Alzheimer’s diseases [25].

Another issue relates to the current food safety assessment scheme that is often conducted under a reductionist one-chemical-one-outcome approach without considering the co-occurrence of multiple chemicals and pesticide exposures. The interplay of chemical mixtures at levels currently considered “safe,” e.g., below the acceptable daily intake, and their effects on human health has recently attracted much attention [28,29]. For example, prenatal exposure to a mixture of 26 suspected endocrine-disrupting chemicals that pregnants may be exposed to via diet or other environmental media was associated with lower levels of cognitive functioning of offspring at age 7 using a mixture-based approach called weighted quantile sum regression [[30], [31]]. Mixture toxicity in the risk assessment process for groups of food contaminants, such as pesticides, PCBs, or metals is not embedded in cohesive and efficient regulatory schemes. Some the reasons for this would be due to mixture analytical challenges including, but not limited to, high rates of type I errors (false positives) or type II errors leading to nondifferential exposure misclassification or multicollinearity due to highly correlated chemical exposures, or positive/negative confounding [28,32].

In addition to the issue of food contaminant mixture effects on human health, biological interactions between nutrients and contaminants in foods at contaminant levels currently considered “safe” and their human health impact shall be revisited. This topic is of interest for poor quality diets or diets where both nutrients and contaminants occur in foods in different mixed ratios. Humans are exposed to mixtures of meals and diets, including their mixtures of nutrients; there are recently some novel approaches providing a holistic assessment of both nutrients and contaminant mixtures in foods and their effects on human health, such as by coupling the use of My Nutrition Index together with a weighted quantile sum index of 26 endocrine-disrupting chemicals [33]. Emphasis is given to a better understanding of whether a good diet would mitigate the impact of food contaminants, such as the scenario of PFAS chemicals in fish and their influence on children’s neurodevelopment or in adult chronic disease risk. For example, the perinatal exposures to PFAS and their association with birth weight were more negative in females with no fish consumption than those pregnant females with any fish consumption [33]. This noteworthy interaction between diet and food contaminant patterns highlights the complexity of dietary patterns and the assessment of their effects on human health.

A nutritionally deficient diet (such as for folic acid, iron, calcium, vitamin A, or ascorbic acid) or an unhealthy dietary pattern, such as the systematic consumption of ultra-processed foods, would influence co-occurring chemical contaminant susceptibility or the chemical intoxication treatment process [34]; for example, lead absorption in infants was enhanced for those experiencing dietary calcium deficiency [34]. In reverse, dietary calcium uptake with foods rich in calcium would reduce gastrointestinal lead absorption as a result of complex biological interactions between lead, dietary calcium, intestinal calcium, binding proteins, and vitamin D [35]. At the population level, dietary shifts from corn to rice/wheat allowed for the reduction in dietary exposures to aflatoxins and led to a ∼10-fold decrease in liver cancer incidence rates for young and middle-aged adults in hepatitis B virus endemic areas of China [36].

## Addressing the Complexity of Food Characterization in the Health Claim Process

In principle, food quality attributes, such as food matrix and composition metrics, dynamically evolve in time, and their stability might be compromised if food contaminants or specific processing patterns enter the food chain at any time point, anywhere from farm-to-fork. There are three important lines of evidence to be presented here in relationship to the health claim process in foods.

Firstly, the often unintentional yet ubiquitous presence of mixtures of chemicals and pesticides in food commodities together with the beneficial (e.g., iodine and PUFA) or detrimental (e.g., sugar or salt content) nutritional attributes. Pesticide residues are a prime example of a ubiquitous food contaminant group historically regulated in foodstuffs and implicated with a series of human health effects. The Farm-to-Fork Strategy, part of the ambitious Green Deal policy, has set concrete targets to transform the EU food system by 2030 toward a more sustainable food future for all, including a reduction by 50% on the use of pesticides and toward reaching 25% of agricultural land under organic farming [3]; the Farm-to-Fork strategy aspires in reconciling the food system toward a more healthy, equitable and environmentally friendly food. A social change to optimize pesticide mixture usage in food crops may be challenging to attain under business as usual because of intertwined requirements for food security, agriculture production, crop management, and environmental protection schemes [37].

Secondly, food composition, and especially its nutritional profiling during the food characterization process of the overall health claim evaluation scheme, is typically considered stable over time as it moves through the farm-to-fork chain of systems. Climate change will likely impact the rates of pest infestation or fungal infection, likely increasing the risk of fungi-based food contaminants, such as aflatoxins, ochratoxin A, and *Fusarium* toxins [38]. The classical notion that the nutritional composition of food commodities is stable may be challenged by new scientific evidence suggesting an atmospheric carbon dioxide (eCO_2_)-associated change in the protein content of foods, but this may considerably vary by commodity class or crop properties [39]. For example, the %eCO_2_-associated change in protein content widely varied from –17.3 (–30.8, –3.8) for vegetables to –0.49 (–2.9, 1.95) for soy [39]. Elevated eCO_2_ concentrations have been projected to increase the risk of protein deficiency with a >7% decrease in protein intake for plant-based diets in countries dependent on staple crops [[6], [7], [5],^39^]. Thus, declining protein and/or micronutrient content of foods would challenge existing health claims of foods based on single nutrients, exhibiting spatiotemporally varying daily intake estimates (mass of nutrient per gram of food consumed) across food commodities and regions. This is an important reason for all countries to maintain updated information on food composition, both spatially and temporally resolved.

Third, co-occurring nutrient-contaminant pairs in foods have not been well characterized, and they are poorly understood in relation to their human health effects. Food contaminant health effects may be modulated by nutrition, serving either as an agonist or antagonist (e.g., ultra-processed foods or foods rich in PUFAs, respectively) [40]. For example, characterizing the food content of folic acid and implementing folate fortification schemes for populations in need would be an effective means of mitigating the adverse health effects of geogenic arsenic via the detoxifying transformation of inorganic to methylated arsenic [41]. On the contrary, excessive folate intake has been associated with masking of vitamin B12 deficiency and metabolic perturbations with vitamin B12 influencing neurocognitive outcomes among the elderly [42].

In other cases, the characterization of co-existing nutrient-contaminant pairs in foods is crucial in benefit-risk assessment exercises; prime examples are the cases of methylmercury and omega-3 fatty acids [43,44], toxic metals and vitamin A [45], hexachlorobenzene (HCB) and vitamin B12, or perfluorooctanesulfonic acid (PFOS) and beta-cryptoxanthin [46]. The interaction of nutrient-contaminant pairs can be illustrated using the example of fish and seafood, which contain the essential PUFAs (*n–*3 PUFA, mainly DHA, 22:6 and EPA, 20:5). The beneficial effects of PUFA on human health would be compromised by the co-occurrence of fish and seafood contaminants, such as PCBs, methylmercury or dioxins, and in some cases, they will be reversed (e.g., in high food contaminant intake groups) [43]. In such a complex yet realistic scenario, where nutrient-contaminant pairs are considered in an epidemiologic study, emphasis shall be paid to the phenomenon of negative confounding; negative confounding is posed by food contaminants that affect in the opposite direction the health outcome when compared with that of the main nutritional exposure of interest, such as methylmercury and PUFAs on neurodevelopmental indices [44]. Common issues of exposure misclassification and negative confounding resulted in the underestimation of fish benefits and methylmercury toxicity [44]. Inverse associations between serum PFAS and serum folate were observed in the NHANES United States survey datasets, suggesting competitive PFAS toxicokinetics as influenced by folate concentrations [45]. High concentrations of vitamin B12 enhanced the magnitude of the association between HCB and the risk of childhood obesity, whereas antioxidant beta-cryptoxanthin and vitamin A protected against the obesogenic effect of food contaminants, such as HCB and lead/nickel chemicals [46]. Certain components of healthy diets, such as fibers, could act as binders for persistent organic chemicals, reducing their absorption [47]. These nutrient-contaminant interactions in foods may be of immense importance during critical life susceptibility windows, such as those of the perinatal period and childhood [48,49]. Accounting for these nutrient-contaminant interactions may be key in better understanding the net beneficial effect of healthy foods/diets on reducing susceptibility to obesogenic effects of food contaminants [50]. The above are prime examples of nutrient-contaminant pairs in foods that are worth studying their interactions and the endogenous response associated with the consumption of such foods by different subpopulation groups. The magnitude and variance of food exposures to such nutrient and contaminant pairs, including their frequency of food consumption, are important factors in facilitating a comprehensive food characterization process as part of a reformed health claim evaluation scheme.

## The Future of Health Claims on Foods

A renewed regulatory framework to allow for updates and revisions in the health claim evaluation process is warranted if we were to support current and future global and EU policies that promote sustainable food production, zero chemical pollution goals, and healthy eating/living in a changing planet, especially for vulnerable sub groups, such as children. The linkages between unhealthy food or poor dietary choices with the NCDs’ burden have been demonstrated in the global burden of disease analyses [11]. Proposed regulatory changes would have to underpin the natural complexity of foods’ content and composition, including their food contaminant content within or across various food categories. For example, wide differences in the magnitude and variance of both nutritional and contaminant profiles have been observed between organic and conventional foods (vitamins, metals, pesticides, bacteria, fungal toxins, and antibiotic resistance metrics) [51].

To address this complexity, the EFSA published a guidance document on the appraisal and integration of evidence from epidemiologic studies for possible use in future scientific assessments [52]. Recent scientific developments in nutritional and environmental epidemiology take on the complexity of co-occurring nutrients and chemical contaminants/residues (e.g., pesticides, lead, etc.) in a wide range of food products/items and how these might be implicated with disease pathogenesis [53,54]. Moreover, novel methodological frameworks that attempt to complement progress in the field of genomics, such as that of the human exposome and its exposome tools, may find use in nutritional epidemiology toward the consideration of multiple dietary exposures by moving away from the exclusive study of single dietary/nutritional components [[55], [56], [57]]. The human exposome methodological framework comprises a holistic approach to the characterization of environmental (nongenetic) exposures and their endogenous response in critical windows of susceptibility, including diet, meals, food products(s) and nutrients, as well as, food contaminants and additives [[55], [56], [57], [58]]. The critical components of exposome-wide association studies in analogy to genome-wide association studies are the following: the multi-method of exposures, correlated exposures and confounding, variable selection challenges, and mixture analysis opportunities and bottlenecks [59].

Such novel approaches would require new study designs and methodological frameworks coupled with the application of advanced mixture-based biostatistical algorithms and data processing pipelines [59]. Major challenges associated with the holistic approach of the human exposome addressing multiple exposure groups and variables moving away from the dogma of one dietary exposure one outcome at a time are: *1*) high dimensionality of exposure data; *2*) joint associations of nutrient-contaminant profiles tackled using novel algorithms (e.g., Bayesian kernel machine regression, quantile g-computation) and 2-way interactions, *3*) -omics platform data integration, and *4*) complex causal structures [60].

## Cumulative Risk Assessment and Risk-Benefit Analysis: Alternative Health Claim Evaluation Processes

At first, critical regulatory backing by the policy makers (e.g., the EU Parliament) would be needed before the relevant authoritative bodies, such as EFSA, undertake alternative health claim evaluation processes. Then, the critical question would be how do we incorporate recent scientific advances in characterizing multiple nutrient-contaminant pairs or contaminant mixtures in foods and their overall health impact(s) into the rigid protocols of evaluating a food health claim application. This type of holistic evaluation may be quite complex, but it has long been recognized within the human nutrition field as a tremendous challenge. One approach would be the assessment of the holistic nature of foods by applying novel cumulative risk assessment (CRA) that incorporates combined risks for food chemical contaminants and pesticides or food additives together with the nutritional attributes of foods. The CRA approach may be particularly useful for sensitive population subgroups (e.g., children) by accounting for multiple chemicals and their chemical and multi-pathway exposure sources via the analysis, characterization, and quantification of the combined risks to health and/or the environment from external agents or stressors [61]. Computational toxicity testing models may also be helpful in identifying those chemicals in food presenting the highest total toxic equivalencies; in a recent exercise, those were chemicals such as food additives, indirect additives, and food-contact substances [62]. Data from the pesticide monitoring survey programs conducted in 10 EU population groups was fed into a pilot CRA exercise on pesticide effects on the thyroid and the nervous system [63,64]. The EFSA has recently published the cumulative dietary exposure assessment for 7 pesticides, studying acute effects on a target organ system (e.g., the nervous system) by combining scientific data on >400 chemicals; this dataset was then incorporated into a cumulative dietary risk assessment of pesticides and their acute effects on the nervous system [65].

Tiered and stepwise approaches for both whole mixture approaches and component-based approaches have been recently promoted by EFSA, and they appear to be on the right track. Special emphasis is paid to grouping chemicals into common assessment groups, using the dose addition as a default assumption, while providing opportunities for reconciling the integration of refined assessment groups and the evidence of interactions [66]. Such an exercise would weigh the positive elements of food against the negative effects of the food contaminants on an organ system. It indeed would become more complicated to conduct such an exercise, especially when the negative elements of food affect different organ systems through different biological mechanisms.

Risk-benefit analysis (or benefit-risk analysis) (RBA) in foods is another approach of merit for possible inclusion in the health claim evaluation process [67,68]. The RBA estimates the overall impact of food on health, integrating both beneficial and adverse effects, and it was first formally discussed by EFSA back in 2006 [69]. Similar to the classical risk assessment model, the RBA approach includes the health effect identification, whether this is a beneficial or adverse effect, followed by exposure assessment, dose-response, and probabilistic risk-benefit characterization, including a proper uncertainty analysis that eventually feeds into risk management and decision making [68,70]. The disability-adjusted life years (DALYs) are commonly used in RBA exercises as a common health metric. When the use of DALYs is not possible, risk ranking techniques for dietary risks/benefits based on multi-criteria decision analysis would be implemented [68]. For example, an RBA weighed the benefits and risks associated with exposure to inorganic arsenic and aflatoxins through the consumption of infant cereals and the risk of developing lung, bladder, and liver cancer over a lifetime [68]. Several RBA case studies have already been published on the risks-benefits of pesticides, the potential of substituting red and processed meat with fish or offering alternatives to bisphenol A [67], including RBAs for weighing in overlapping micronutrients (folate, niacin, selenium, fluoride) risks and benefits [71]. A case study of RBA on substituting red and processed meat with fish considered the following health outcomes associated with fish consumption: protection against fatal coronary artery disease (DHA and EPA), enhanced neurodevelopment (fish), compromised neurodevelopment (methylated mercury, MeHg), thyroid toxicity (dioxin and dl-PCBs), and male infertility toxicity (dioxin and dl-PCBs), whereas the considered health outcomes associated with consumption of red and processed meat were: colorectal cancer (red and processed meat), stomach cancer (processed meat), thyroid toxicity (dioxin and dl-PCBs), and male infertility toxicity (dioxin and dl-PCBs) [67]. The RBA results showed that the main drivers of the overall health impact were the beneficial effects of PUFAs in fish on fatal coronary artery disease and the effect of fish consumption (beneficial and adverse) on neurodevelopment in unborn children [67]. Approximately 150 DALYs/100,000 of the population could be averted each year if adults consumed 350 g of fish/wk (fatty or mix of fatty and lean) while decreasing the consumption of red and processed meat [67]. The RBA health impact of the substitution was depicted in the DALYs difference between the alternative scenario and the current consumption by health outcome, showing that the substitution of red and processed meat with fish is overall beneficial with 5786 (95% uncertainty interval: 4390; 7299) DALYs averted each year for the Danish population (15–75 y of age) [72].

### Implications for the future of health claims

**FIGURE 1:**
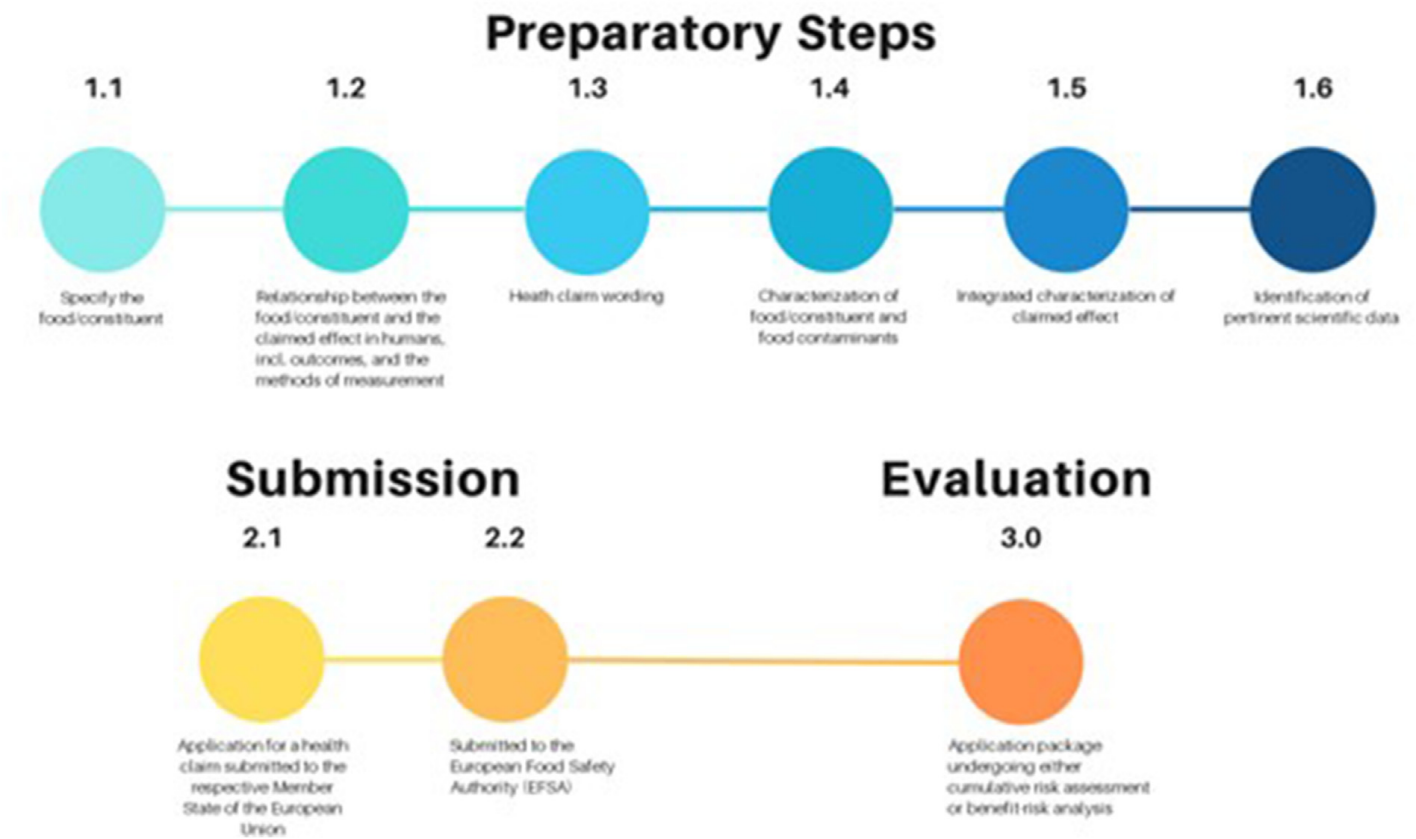
The overall health claim evaluation process for foods, including the various stages, ranging from preparatory steps to submission of the health claim application package and its evaluation. The major reforms of the current health claim evaluation process would be highly relevant for stages 1.1, 1.4, and 3.0.

Focusing solely on food safety aspects with a reductionism strategy, this approach alone may not always protect consumer health. Food may also impact human health and well-being for reasons that fall outside the scope of food safety. As proposed in this work, a reform in the health claim evaluation process for foods may be supported by considering the incorporation of both CRA and RBA approaches into the food characterization and food-health stages of the evaluation process (Figure 1). All in all, a revised CRA exercise for foods shall focus on a specific organ system, health outcome, or biological mechanism, allowing for the proper evaluation of pertinent health claims. The RBA approach could also find use in the health claim evaluation process for multiple nutrient-contaminant pairs or for food contaminant/nutrient or food mixtures and their effects on various health outcomes. The consideration of such alternative methodological frameworks and health impact assessment processes would tell us whether traditional foods or new foods with strong holistic nutritional properties negate the adverse effects of present chemical contaminants in that food and vice versa. That may be of immense importance for sensitive population subgroups, such as children, pregnant females, or the elderly.

Overall, a more holistic assessment of food characterization in health claims by comprehensively combining multiple nutritional properties and contaminant pairs and/or mixtures in food and their integration with a CRA or RBA methodology shall be promoted (Figure 1). It is due time for policymakers and the food industry to revisit regulations for the evaluation of health claims that have been in place for decades. The CRA and RBA approaches, including the computational toxicity prioritization models, are options available for wider use, given the accumulating evidence of food-based contaminant mixture effects on human health, including the biological and statistical interactions of chemical contaminants with nutritional food attributes. An alternative framework of risk assessment, food characterization, and risk management abiding by the principles of CRA and RBA would be timely to consider together with the regulatory bodies of the health claim regulatory system. Such reforms in the health claims policy in foods would potentially “spill over” to the general food law toward dealing with the gray area foods (e.g., ultra-processed foods) that are currently considered “safe” on the one hand, but their systematic consumption patterns would potentially lead to the development of chronic diseases in the long-term. Inevitably, such reforms would support and promote the better implementation of the global and EU strategies of the Zero Pollution Action Plan, the Farm-to-Fork, the Green Deal, and the SDGs (e.g., targets 2.4 and 3.4).

## Author contributions

The authors’ responsibilities were as follows – KCM: was responsible for design, writing, and final content; MC: contributed to the critical revision of the manuscript. Both authors read and approved the final manuscript.

### Conflict of interest

The authors report no conflicts of interest.

## Funding

The authors reported no funding received for this study.
